# A *Brucella* spp. Isolate from a Pac-Man Frog (*Ceratophrys ornata*) Reveals Characteristics Departing from Classical Brucellae

**DOI:** 10.3389/fcimb.2016.00116

**Published:** 2016-09-28

**Authors:** Pedro F. Soler-Lloréns, Chris R. Quance, Sara D. Lawhon, Tod P. Stuber, John F. Edwards, Thomas A. Ficht, Suelee Robbe-Austerman, David O'Callaghan, Anne Keriel

**Affiliations:** ^1^Institut National de la Santé et de la Recherche Médicale, U1047, UFR de MédecineNîmes, France; ^2^Université de Montpellier, U1047Nîmes, France; ^3^Mycobacteria and Brucella Section, National Veterinary Services Laboratories, Animal and Plant Health Inspection Service, United States Department of AgricultureAmes, IA, USA; ^4^Clinical Microbiology, Department of Veterinary Pathobiology, College of Veterinary Medicine and Biomedical Science, Texas A&M UniversityCollege Station, TX, USA; ^5^Department of Veterinary Pathobiology, Texas A&M UniversityCollege Station, TX, USA

**Keywords:** *Brucella*, frog, motility, metabolism, LPS, virulence, evolution, *Ochrobactrum*

## Abstract

*Brucella* are highly infectious bacterial pathogens responsible for brucellosis, a frequent worldwide zoonosis. The *Brucella* genus has recently expanded from 6 to 11 species, all of which were associated with mammals; The natural host range recently expanded to amphibians after some reports of atypical strains from frogs. Here we describe the first in depth phenotypic and genetic characterization of a *Brucella* strains isolated from a frog. Strain B13-0095 was isolated from a Pac-Man frog (*Ceratophyrus ornate*) at a veterinary hospital in Texas and was initially misidentified as *Ochrobactrum anthropi*. We found that B13-0095 belongs to a group of early-diverging brucellae that includes *Brucella inopinata* strain BO1 and the *B. inopinata*-like strain BO2, with traits that depart significantly from those of the “classical” *Brucella* spp. Analysis of B13-0095 genome sequence revealed several specific features that suggest that this isolate represents an intermediate between a soil associated ancestor and the host adapted “classical” species. Like strain BO2, B13-0095 does not possess the genes required to produce the perosamine based LPS found in classical *Brucella*, but has a set of genes that could encode a rhamnose based O-antigen. Despite this, B13-0095 has a very fast intracellular replication rate in both epithelial cells and macrophages. Finally, another major finding in this study is the bacterial motility observed for strains B13-0095, BO1, and BO2, which is remarkable for this bacterial genus. This study thus highlights several novel characteristics in strains belonging to an emerging group within the *Brucella* genus. Accurate identification tools for such atypical *Brucella* isolates and careful evaluation of their zoonotic potential, are urgently required.

## Introduction

*Brucella* are highly infectious bacterial pathogens responsible for brucellosis, a frequent worldwide zoonosis (500,000 new cases/year). Infection is usually transmitted to humans by direct contact, ingestion of contaminated animal products or aerosolization (De Figueiredo et al., 2015). The disease is mainly characterized by abortion or sterility in animals, while in humans it is a serious febrile disease with varied symptomatology including undulant fever, headaches, muscle pain, night sweats, asthenia, and depression (Moreno, 2014). Infection often requires hospitalization and at least 6 weeks of antibiotic treatment; if left untreated, patients can develop serious complications such as neurobrucellosis or endocarditis.

*Brucella* are Gram-negative coccobacilli belonging to the family brucellaceae together with *Ochrobactrum* spp., their closest phylogenetic relatives. This family belongs to the order Rhizobiales, which contain several genera of plant-associated alpha proteobacteria. The *Brucella* genus is currently composed of 11 species, some of them considered to be pathogenic to humans. The classification of the six “historical” species is based on phenotypic characteristics and each has a preferential host: *Brucella melitensis* (goats), *Brucella suis* (suidae), *Brucella abortus* (cows), *Brucella ovis* (sheep), *Brucella canis* (dogs), and *Brucella neotomae* (desert rats) (Moreno, 2014). Over the past few decades strains isolated from marine mammals have been included in the genus as *Brucella ceti* (cetaceae) and *Brucella pinnipedialis* (pinnipeds) (Foster et al., 2007). The most recently identified species are *Brucella microti* (voles, Audic et al., 2009), *Brucella inopinata* (a human breast implant infection, Scholz et al., 2010), *Brucella papionis* (stillbirth in baboons, Whatmore et al., 2014), and *Brucella vulpis* (red foxes, Scholz et al., 2016). Other atypical *Brucella*-like strains have not been assigned a specific designation; *B. inopinata*-like strain BO2 (chronic pneumonia in a human) (Tiller et al., 2010a) and strains isolated from wild rodents in Australia (Tiller et al., 2010b). Isolation of *Brucella* strains from non-mammalian hosts is exceptional, however three reports recently described the isolation of atypical *Brucella* strains from amphibians: a Big-eyed tree frog (*Leptopelis verniculatus*, Fischer et al., 2012), several African bullfrogs (*Pyxicephalus edulis*, Eisenberg et al., 2012) and a White's tree frog (*Litoria caerulea*, Whatmore et al., 2015).

*Brucella* are facultative intracellular pathogens that can evade the innate defense mechanisms and reach an intracellular replication niche comprised of vesicles derived from the endoplasmic reticulum (Celli, 2015). Intracellular replication of *Brucella* requires a type IV secretion system (T4SS), an apparatus used by many bacterial pathogens to deliver effectors into host cells and that is considered as a major virulence factor in *Brucella* (Celli, 2006; Lacerda et al., 2013). It also depends on the chemical structure of their lipopolysaccharide (LPS, Porte et al., 2003; Mancilla, 2016). LPS is composed of an O-polysaccharide (O-PS, or O-antigen), a long chain formed by one or two sugar repeats, lipid A, which anchors LPS in the outer membrane, and a short core oligosaccharide (OS) that serves as a linker between lipid A and O-PS. Other than two naturally occurring rough species (*B. canis* and *B. ovis*) there is little variability in the composition and structure of the LPS within the *Brucella* genus (Lapaque et al., 2005). Exceptions to this rule were described for the recently isolated *B. inopinata* strain BO1 and strains isolated from Australian wild rodents, which appear to have modification in the LPS core, and for strain BO2 which produces an atypical O-PS (Wattam et al., 2012; Zygmunt et al., 2012).

Here, we report the first case of *Brucella* infection of an amphibian in America, confirming that these *Brucella* are found throughout the world. This strain (B13-0095) was isolated from a Pac-Man frog (*Ceratophyrus ornate*) in a veterinary hospital in Texas. It was initially misidentified as *Ochrobactrum anthropi*, but belongs to an emerging clade of atypical *Brucella* that includes the strains BO1 and BO2 (“BO clade”). Analysis of the B13-0095 genome led to the identification of new metabolic pathways shared with *O. anthropi*, several specific to B13-0095, and others that are conserved within the BO clade. We also found that this strain produces and atypical O-PS and has a faster intracellular replication rate than classical *Brucella* spp. Finally, we demonstrate that the BO clade *Brucella* strains exhibit motility, a phenotype not described for this bacterial genus.

## Materials and methods

### Bacterial strains

Bacterial strains used in this study are listed in Table 1. Upon primary isolation and characterization, the Pac-Man frog *Brucella* isolate was cultured onto trypticase soy (TS) agar plates supplemented with 5% (v/v) sheep's blood (BAP) (Becton Dickinson; Franklin Lakes, NJ) and incubated at 37°C in an atmosphere supplemented with 5% (v/v) carbon dioxide. Slants of agar media (Becton Dickinson; Franklin Lakes, NJ) inoculated with the organism were incubated overnight at 37°C in an air atmosphere. For further experiments, all strains were cultured on TS medium at 37°C. Fluorescent derivatives of the Pac-Man frog isolate (bIN1996) or of *B. suis* 1330 (bIN1505) were obtained by electroporation with pMR10 based plasmid encoding mCherry.

**Table 1:** **List of bacterial strains used in this study**.

**Strain name**	**Description (reference)**
B13-0095	*Brucella* isolate from a Pac-Man frog (this study)
1330	*Brucella suis* biovar 1 type strain, ATCC 23444^T^
1330ΔVirB8	ΔVirB8 mutant in *B. suis* 1330 (Paschos et al., 2006)
16M	*Brucella melitensis* biovar 1 type strain, ATCC 23456^T^
2308	*Brucella abortus* biovar 1
BO1	*Brucella inopinata* type strain, isolated from an infected breast implant in a 71-year-old patient from Oregon, USA (Scholz et al., 2010)
BO2	*B. inopinata*-like isolate, isolated from a 52-year-old Australian patient with chronic destructive pneumonia (Tiller et al., 2010a)
CCM4915	*Brucella microti* strain BCCN 07-01; isolated from infected voles in the Czech Republic (Scholz et al., 2008b)
NF2653	Representative of atypical *Brucella* strains isolated from wild rats in Queensland, Australia (Tiller et al., 2010b)
*O. anthropi* ATCC49188	*Ochrobactrum anthropi* type strain

### *Brucella*-specific PCR

PCR was performed using primers Bruc-F (5′-AACCACGCTTGCCTT GCACACC-3′) and Bruc-R (5′TTTCAAGCGCCTGTT CACCCG-3′) (Scholz et al., 2008a). The PCR reaction included 2.5 mM MgCl_2_, 2.0 mM dNTPs, 150 nM of each primer, and 0.5 U AmpliTaq DNA Polymerase (Applied Biosystems; Foster City, CA).

### Genome analysis

Whole genome sequencing (WGS) was performed as described recently (Quance et al., 2016). Phylogenetic placement was performed using kSNP, a program that can analyze raw, unassembled reads (Gardner and Hall, 2013) using the genomes listed in Table S1. Genome assembly and automatic annotation was performed using the PATRIC resource^1^ (Wattam et al., 2014a). Confirmation of the presence of predicted protein encoding genes in other genomes was performed using BLASTP^2^ within the *Brucella* genus (Taxonomic ID 234) or *Brucellaceae* family (Taxonomic ID 118882). A gene was considered to be present and conserved when the identity of the protein sequences were ≥90% within the *Brucella* genus. When comparing *Brucella* with *Ochrobactrum* we used ≥75% over ≥95% of the sequence as a cut off for presence of a homologe.

### Use of different carbon sources

Bacteria were grown in a chemically defined medium as previously described (Barbier et al., 2014). Briefly, bacteria were pre-cultured in a modified Plommet medium containing erythritol (2 g/L) for 16 h, washed with PBS and adjusted to an OD_600_ of 0.02 in modified Plommet containing either erythritol, ectoine (20 mM), or L-rhamnose (1 g/L). Growth was measured by reading the OD_600_ after 24 h of culture.

### Motility assays

In tube motility assays were performed in Difco™ Motility GI Medium (Becton Dickinson) supplemented with 0.05 g/L 2,3,5-triphenyltetrazolium chloride (TTC) to help visualization of motility, according to the manufacturers recommendations. On plate swimming assays were performed as previously described (Ha et al., 2014). Briefly, TS plates containing 0.25% (w/v) agar were inoculated with a 5 μl drop of bacterial suspension at an OD_600_ of 1 at their center and motility was followed macroscopically for up to 5 days. Each condition was tested in independent experiments at least three times. Motility surface was measured using Image J^3^.

### LPS analysis

Extraction of LPS with SDS-proteinase K was performed as previously described (Soler-Lloréns et al., 2014). Extracts were separated on 11% (w/v) polyacrylamide gels and stained using the periodate-alkaline silver method (Tsai and Frasch, 1982). The reaction was stopped with 1% (v/v) acetic acid and pictures were taken with a Panasonic Lumix DMZ-6 camera.

### Intracellular replication profiles

Human epithelial (HeLa) and murine macrophage cells (J774) were infected using gentamycin protection assays with an MOI of 500 or 50, respectively as described previously (Keriel et al., 2015). At 2, 5, 24, and 48 h post-infection (hpi), cells were lysed with 0.1% Triton X-100 (v/v) for 10 min. Serial dilutions of the cell lysates were then plated on TS plates and colonies were counted after 1 or 2 days of incubation at 37°C.

### Microscopy analysis

Cells were plated on sterile glass coverslips, infected as described above and fixed with paraformaldehyde (3% (w/v) PFA, 25 min, RT) at 24 hpi. Slides were mounted using ProLong gold media (Invitrogen) and visualized using a confocal microscope (Fluoview Fv10i, Olympus). For low magnification visualization, non-confocal images were acquired at a 1024 × 1024 resolution using a 10x objective (NA 0.4). Confocal images were acquired with a 1024 × 1024 resolution using the XY mode of the confocal microscope and a 60x oil immersion objective (NA 1.35).

### Flow cytometry analysis

Cells were infected as above with the exception that mCherry-expressing bacteria were used. At 24 hpi cells were detached with trypsin, fixed, and analyzed by flow cytometry (MACSQuant VYB, Miltenyi). Data were analyzed using the MACSQuantify software. This gave % of infected cells (i.e., % of mCherry positive cells) as well as the MFI (median fluorescence intensity) of infected cells, which is indicative of the number of bacteria inside cells.

## Results

### Case report

A six-year-old female, 192 g, reticulated Pac-Man frog (*Ceratophrys ornata*) was presented to the Texas A&M University Veterinary Medical Teaching Hospital (College Station, TX, USA) in February 2010 for depression, anorexia, weight loss, and a history of ingestion of foreign bodies (animal ID: 191570).

Five months prior to presentation, the frog ingested small glass beads that were part of the decorations in her enclosure, and was then moved to a different enclosure that contained a moss substrate. The following month, the frog ate an adult mouse and passed normal feces. Subsequently, the frog did not eat, despite the multiple attempts to feed her, and did not pass feces. The owner reported that the live prey used to feed the frog traumatized its digits and back. Prior to ingesting the foreign bodies, the frog had no history of illness. This pet was fed with a diet of live mice and received no additional nutritional supplements. A natural sunlight lamp was used to illuminate the frog's enclosure, and it was covered at night with a towel. The ambient temperature of the enclosure was maintained between 21–24°C.

On presentation, the frog was unresponsive with fixed and dilated pupils. No pupillary light response was noted. Three to five, glass beads were palpable within the coelomic cavity. The distal phalanges of digits 3 and 4 of the right rear leg were missing, and skin over the back was eroded. The frog was determined to be 5–10% dehydrated. No other apparent abnormalities were noted on physical exam. The frog had a body condition score of 1 out of 5. Because of a poor prognosis for recovery, and after informed consent of the owner, the frog was humanely euthanized by first anesthetizing it with a tricaine methanesulfonate (MS-222) bath followed by intracoelomic, pentobarbital sodium injection. Euthanasia was confirmed via Doppler ultrasound.

At necropsy, the liver was tan and friable, and the coelomic cavity of the frog had numerous strands of fibrin and firm adhesions adhering the coelomic organs to each other and to the body wall indicating subacute to chronic peritonitis. The stomach contained six, 2 × 1 cm, elliptical, smooth-surfaced glass beads, but grossly, they did not appear to have affected the stomach. No evidence of metabolic bone disease was observed. No significant lesions were observed in the other body systems. Histologic examination of the liver indicated severe, diffuse, vacuolar degeneration with intrahistiocytic pigment. The final diagnosis was severe, chronic peritonitis. Samples of the liver and peritoneum were submitted for bacterial culture.

### Bacterial isolation, phenotypic characterization and identification

The liver and peritoneum specimens were inoculated onto culture plates. Following overnight incubation, pinpoint, alpha-hemolytic bacterial colonies grew from the liver (culture 10060005-1), and peritoneum (culture 10606004-1). Slants of triple sugar iron (TSI) agar, lysine iron agar (LIA), urea agar, Simmons' citrate agar and tryptophan broth were inoculated with the organism. The organism was oxidase and urease positive, and did not utilize citrate or produce indole from tryptophan. On the TSI and LIA slants, the isolate did not ferment lactose or sucrose and did not produce hydrogen sulfide.

An API 20NE identification kit (bioMérieux) performed on the peritoneum isolate yielded identification codes of 0241324 at 24 h and 0243334 at 48 h (Table 2). When compared with the API online database (https://apiweb.biomerieux.com), this isolate was identified as *O. anthropi* with a probability of 99.9%. However, some results were not consistent with this identification such as the positive adipic acid assimilation, the negative malate assimilation and the absence of nitrate reduction at 48 h. Literature searches were performed at PubMed using various combinations of the search terms of “*Ochrobactrum*,” “*Brucella*,” “frog,” and “amphibian.” No case reports indicating prior isolation of *Brucella* species from frogs were found so no further efforts to identify the organism were made. Because of the unusual nature of the case, the isolates were stored in 10% glycerol at −80°C.

**Table 2:** **API^®^ 20 NE Biochemical test results at 24 and 48 h for the Pac-Man frog *Brucella* isolate B13-0095**.

**Test**	**24 h**	**48 h**
Reduction of nitrates to nitrites or nitrogen	−	−
Indole production	−	−
Fermentation of glucose	−	−
Arginine dihydrolase	−	−
Urease	+	+
Hydrolysis of esculin	−	−
Hydrolysis of gelatin	−	−
β-galactosidase production	−	−
Glucose assimilation	+	+
Arabinose assimilation	+	+
Mannose assimilation	**-**	+
Mannitol assimilation	−	−
N-acetyl-glucosamine assimilation	+	+
Maltose assimilation	+	+
Potassium gluconate assimilation	−	−
Capric acid assimilation	−	+
Adipic acid assimilation	+	+
Malate assimilation	−	−
Trisodium citrate assimilation	−	−
Phenyl acetic acid assimilation	−	−
Cytochrome oxidase	+	+

Several years after the original isolation of the organism, the literature search was repeated and yielded a report of isolation of a potentially novel *Brucella* species from frogs (Eisenberg et al., 2012) along with a case report documenting abscesses associated with a *B. inopinata*-like bacterium in a big-eyed tree frog purchased from a pet store (Fischer et al., 2012). The two isolates were thus revived and a *recA* gene-based multi-primer PCR was performed. Both yielded a 167 bp product, specific to the *Brucella* genus (data not shown). An attempt was made to characterize the liver isolate (thereafter referred as B13-0095) using traditional *Brucella* biochemical analysis (Alton et al., 1988) at the National Veterinary Services Laboratories (NVSL, Ames, IA, USA). The isolate did not conform to the characteristics of any recognized *Brucella* species. The unusual profile included strong urease activity, no requirement for carbon dioxide, no production of hydrogen sulfide, no sensitivity to the dyes thionin or basic fuchsin at 1:25,000, no agglutination with monospecific anti-A and M serum, and no lysis by Tbilisi phage at RTD or RTD × 10^4^. B13-0095 was later analyzed by matrix-assisted laser desorption/ionization time-of-flight (MALDI-TOF) mass spectrometry, using a Vitek MS and a database able to identify *Brucella* isolates at the species level (J. Mesureur and A. Keriel, unpublished data). B13-0095 was clearly discriminated from *O. anthropi* and was identified as *B. inopinata* with an identification score ≥99.99%.

B13-0095 has an atypical growth rate for *Brucella*, as it gives 2–3 mm large colonies within 48 h at 37°C on blood-agar plates. We quantified its growth rate in liquid broth, compared to *B. suis* 1330 as a representative of classical *Brucella* species. B13-0095 reached stationary phase within 5 h of culture, whereas *B. suis* required at least 24 h to do so (data not shown), confirming that B13-0095 has a very rapid growth *in vitro*.

### Analysis of the Pac-Man frog isolate genome

**Figure 1:**
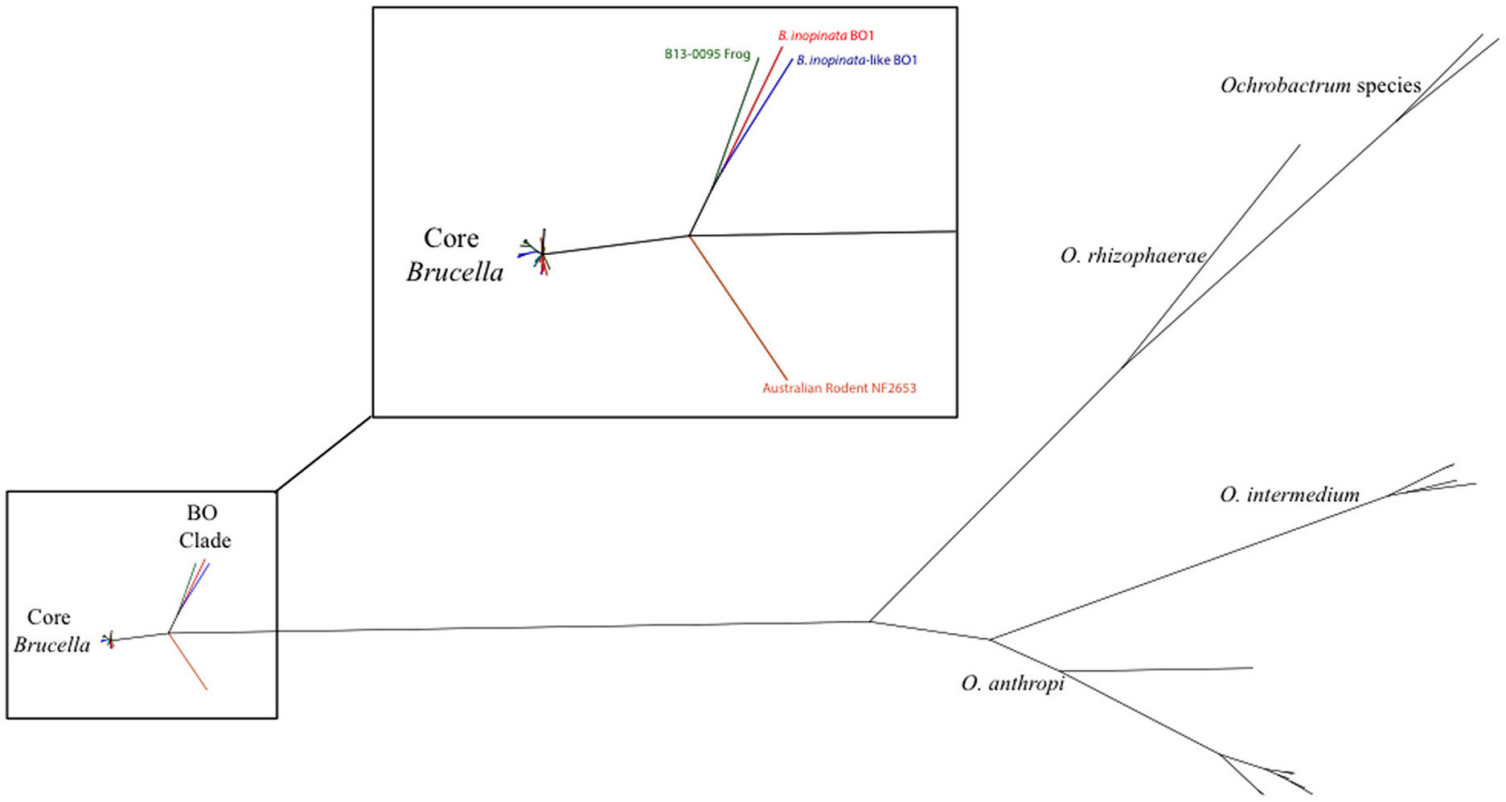
**Phylogenetic placement of B13-0095 relative to other *Brucella* spp. and *Ochrobactrum* spp. determined by kSNP**. The reference strains for all classical *Brucella* spp. and novel species were included. The magnified region highlights the divergence point of the “BO clade” in *Brucella*.

Whole genome sequencing (WGS) was performed on B13-0095 at the NVSL. Analysis shows the presence of the IS711 insertion, a genetic hallmark of *Brucella*. A phylogenetic tree was drawn using kSNP (Gardner and Hall, 2013) with “core” *Brucella* spp., comprising the classically described pathogenic species, and some recently described “atypical” *Brucella* strains (Figure 1). B13-0095 clearly fell within a clade containing the *B. inopinata* strain BO1 and the *B. inopinata*-like isolate BO2 that have been previously described (Wattam et al., 2012). This group, further referred as the BO clade, was very close to strain NF2653, a representative of Australian wild rodents isolates, which is consistent with previous findings (Wattam et al., 2012). This clade is also less distant from *Ochrobactrum* than the classical *Brucella* spp. Full sequence alignment of the 16S rRNA gene of B13-0095 with sequences of BO1, BO2, the *Brucella* spp. consensus sequence and *O. anthropi* showed that B13-0095 shares 100% sequence identity to those of BO1 and BO2, 99.6% identity with other *Brucella* spp. and 97.7% with *O. anthropi* ATCC 49188. The draft sequence was deposited in NCBI under the accession number SAMN05277611. The FASTQ files were deposited in NCBI Short Read Archive under the bioproject number PRJNA326393.

B13-0095 genome was then assembled, resulting in 38 contigs (338 bp to 767 kbp). Automatic annotation using RAST predicted 3326 protein encoding genes (peg), and PATRIC FigFam search identified 20 that seem unique to B13-0095 among the *Brucella* genus (Table 3) and 26 that are conserved within the BO clade, among which 10 are also present in *O. anthropi* genome (Table S2). Notably, the genes of the *virB* operon, which encode the components of *Brucella* T4SS, are present in the genome of B13-0095 (from peg152 to peg142, annotated as VirB1 and VirB11, respectively).

**Table 3:** **List of protein encoding genes found to be specific the *Brucella* strain B13-0095 among the sequenced *Brucella* genomes using a FigFam search in PATRIC.**^4^

**peg #**	**Annotation (in PATRIC)**
1508	L-2,4-diaminobutyric acid transaminase DoeD (EC 2.6.1.-)
1510	DNA-binding protein DoeX, ectoine utilization regulator
1512	Ectoine utilization protein EutB, threonine dehydratase-like
1513	Ectoine utilization protein EutC, similar to ornithine cyclodeaminase
1514	Ectoine hydrolase
1515	N-alpha-acetyl-L-2,4-diaminobutyrate deacetylase
1516	Ectoine/hydroxyectoine ABC transporter solute binding protein, EhuB
1517	Ectoine/hydroxyectoine ABC transporter permease protein, EhuC
1518	Ectoine/hydroxyectoine ABC transporter permease protein, EhuD
1519	Ectoine/hydroxyectoine ABC transporter ATP-binding protein, EhuA
1520	Transcriptional regulator GabR of GABA utilization (GntR family with aminotransferase-like domain)
2599	hypothetical protein
3149	Phage protein, HK97, gp10
3157	hypothetical protein
3227	Recombinase
3230	Mobilization protein MobA/Conjugal transfer protein TraA
3263	Lysine-epsilon oxidase (EC 1.4.3.20) antimicrobial protein LodA
3267	Transposase and inactivated derivatives
3268	Type III restriction enzyme, res subunit:DEAD/DEAH box helicase, N-terminal
3283	DNA-cytosine methyltransferase (EC 2.1.1.37)

**Figure 2:**
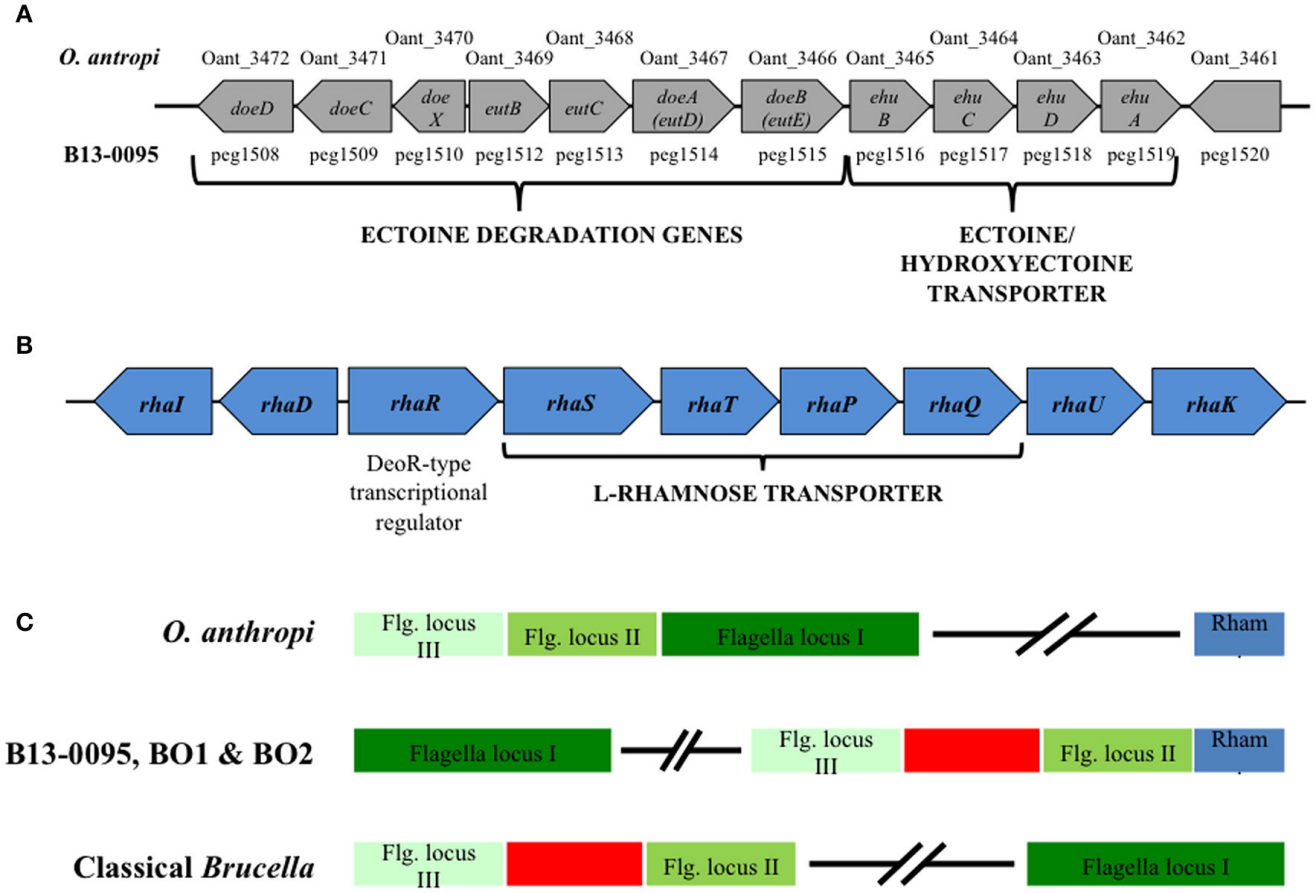
**Genetic clusters specific to B13-0095, BO1, and BO2. (A)** Schematic representation of a 12.5 kb ectoine utilization genetic cluster conserved in B13-0095 and *O. anthropi*. **(B)** Schematic representation of the genes organization in a cluster involved in rhamnose utilization that is conserved in the B13-0095, BO1, BO2, and *O. anthropi*. **(C)** Position of the cluster of rhamnose utilization (in blue) relatively to the three loci of flagella related genes (in green) in the BO clade *Brucella*, compared to *O. anthropi* and classical *Brucella*. The red box represents a fragment of 14 kb containing genes involved in several metabolic pathways or cationic antimicrobials resistance that is present in all *Brucella*.

The genes highlighted in gray belong to the ectoine utilization cluster described in Figure 2A.

### An ectoine catabolic pathway unique to the Pac-Man frog isolate among the *Brucella* genus

Among the unique features in B13-0095 genome, we found that a 12.5 kb fragment encompassing 11 genes highly conserved between B13-0095 and *O. anthropi* ATCC49188. Most of these genes are involved in ectoine uptake (through an ABC-type transporter) or degradation (Figure 2A). The gene *doeC* (encoding an aspartate-semialdehyde dehydrogenase) is present in this cluster, but was not listed as a B13-0095 specific gene (Table 3) because it is conserved in several other *Brucella* spp. We found none of the genes required for ectoine/hydroxyectoine (*ectABCD*) biosynthesis in the B13-0095 or *O. anthropi* genomes, suggesting that these bacteria cannot synthesize (hydroxy)ectoine *de novo*.

Ectoines (ectoin and hydroxyectoin) are water-soluble organic compounds produced by many halophilic bacteria. They protect these microorganisms from deleterious environmental conditions, such as osmotic, thermal, or hygrometric stress (Oren, 2008), however, neither B13-0095 nor *O. anthropi* showed thermo- or osmo-tolerance compared to BO1 and BO2, and exogenously provided ectoine did not restore the growth rate of B13-0095 under hyperosmotic stress (data not shown). Alternatively, ectoines can be used as carbon and nitrogen sources. To ascertain whether B13-0095 can do so, we tested its ability to grow in a chemically defined medium with ectoine as sole carbon source. While all *Brucella* species tested could use erythritol, only B13-0095 was able to use ectoine as a sole carbon source (Table 4), demonstrating a new catabolic pathway that is specific to B13-0095 among the *Brucella* genus.

**Table 4:** **Carbon sources in *Brucella* sp. B13-0095, BO1, and BO2**.

	**Growth in Plommet media**			
**Isolate**	**No carbon source**	**Erythritol**	**Ectoine**	**Rhamnose**
*O. anthropi*	− (0.05)	+ (0.96)	+ (0.53)	+ (1.12)
B13-0095	− (0.03)	+ (1.03)	+ (0.64)	+ (0.98)
BO1	− (0.03)	+ (1.03)	− (0.03)	+ (0.92)
BO2	− (0.03)	+ (1.16)	− (0.03)	+ (1.07)
NF2653	− (0.03)	+ (0.66)	− (0.03)	− (0.03)
*B. suis* 1330	− (0.03)	+ (0.57)	− (0.03)	− (0.02)

Carbon source usage was determined by the ability of the indicated strains to grow in a chemically defined medium containing either ectoine or rhamnose as a sole carbon source. Erythritol was used as a positive control. + = growth, − = no growth. Values in parenthesis indicate the OD_600_ after 24 h of culture (except for O. anthropi for which growth was measured after 48 h).

### A rhamnose catabolism pathway unique to the BO clade *Brucella*

Among the genes conserved within the BO clade *Brucella* and *O. anthropi*, several genes, arranged as a cluster, are involved in L-Rhamnose (rhamnose) uptake and catabolism (Table S4). Rhamnose is a sugar commonly found in nature; it is a constituent of the cell wall of some plants and a potential carbon source for plant-associated bacteria (Eagon, 1961). Its catabolism also plays a significant role in some bacteria-plant interactions, e.g., nodulation of rhizobia (Oresnik et al., 1998).

The genes found in this locus, conserved in B13-0095, BO1, BO2, and O. *anthropi*, were predicted to encode a set of rhamnose catabolic enzymes (RhaI, RhaD, and RhaK), an ABC-type rhamnose transport system (RhaS, RhaT, RhaP, and RhaQ) and a DeoR-type transcriptional regulator (RhaR) (Figure 2B). The gene encoding a rhamnose mutarotase (RhaU) was not identified through the PATRIC FigFam search, but a BLASTN search confirmed that this gene is indeed present in the genomes of B13-0095, BO1, BO2, and *O. anthropi* (peg.238, WP_008511386.1, WP_009363826.1, and WP_012092962.1 respectively), but not in any other brucellaceae. In B13-0095 and the BO clade *Brucella*, as well as in *O. anthropi*, the genes *rhaRSTPQUK* are oriented divergently from *rhaDI.* In *O. anthropi* this genetic cluster is very distant from the genes encoding the flagella apparatus, which are arranged as 3 successive loci (Figure 2C and Table S3). In *Brucella* genomes however, the 3 flagella loci are separated and contain, between flagella loci II and III, a genomic fragment of 14 kb including genes involved in several metabolic pathways (lipid or carbohydrates) or in resistance to cationic antimicrobials. In the BO clade of *Brucella* however, the rhamnose locus is located in the immediate vicinity of the flagella locus II.

Using chemically defined media, we found that B13-0095, BO1, BO2, and *O. anthropi* can use rhamnose as a carbon source (Table 4), highlighting a new catabolic pathway specific to this group of *Brucella*.

### The *Brucella* BO clade are motile bacteria

**Figure 3:**
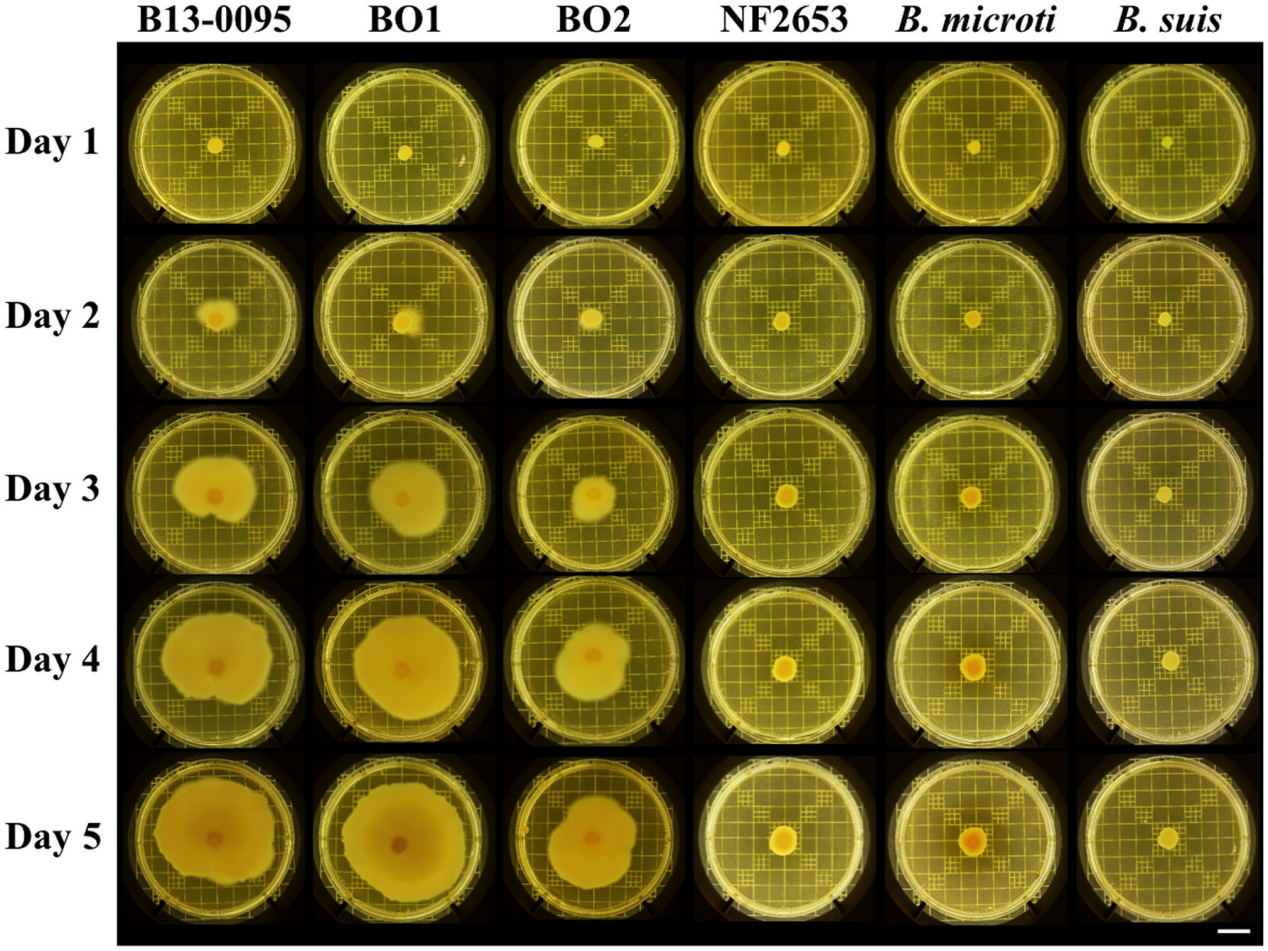
**Motility of *Brucella* strains**. Motility of the indicated strains was visualized using on plate swimming assays. Shown are representative results of at least 3 independent repeats. Scale bar = 2 cm.

*Brucella* are historically classified as non-motile, however Eisenberg et al. (2012) recently reported that *Brucella* isolates from African bullfrogs were motile. Using swimming motility assays on soft-agar plates, we found that B13-0095 is also highly motile, the bacterial layer covering a surface of up to 28 cm^2^ in 5 days (Figure 3). Interestingly, BO1 and BO2 are also highly motile, while NF2653 (closest relative to the BO clade), *B. microti* (a non-related, fast-growing strain) and *B. suis* (classical *Brucella*) are non-motile.

Although non-motile, the genomes of the classical species contain all the genes necessary to assemble an active flagellum, however several are pseudogenes (Letesson et al., 2002; Chain et al., 2005). Analysis of all flagella related genes previously predicted to be pseudogenes in either *B. melitensis* 16M, *B. suis* 1330, or *B. abortus* 2308 (Chain et al., 2005) confirmed the absence of pseudogenes in B13-0095 or BO1 and BO2 (Table 5). However, the genes encoding the flagellar biosynthesis protein FlhA and the flagellar M-ring protein FliF are both pseudogenes in strain NF2653, which was not motile in our assays. This suggests that the BO clade *Brucella*, including B13-0095, may be able to assemble functional flagella, explaining their motility.

**Table 5:** **Analysis of the flagella related genes in the BO clade *Brucella***.

**Flagella locus**	**Gene symbol**	**Protein name**	**Function**	**Accession#**			**PATRIC ID (peg#) and % Identity**			
				***B. melitensis 16M***	***B. suis* 1330**	***B. abortus 2308***	**B13-0095**	**BO1**	**BO2**	**NF2653**
I	*flhA*	Flagellar biosynthesis protein FlhA	Export apparatus	BMEII0166/0167	BRA1132	BAB2_1091/1090/1089	14	2218 (99%)	1188 (99%)	3176/3177
	*motC*	Chemotaxis protein MotC	Motor	BMEII0155	BRA1143	BAB2_1102	25	2229 (98%)	1177 (98%)	3188 (98%)
	*fliF*	Flagellar M-ring protein FliF	MS ring component	BMEII0151/0152	BRA1146	BAB2_1105	28	2232 (99%)	1174 (99%)	3191/3192
II	*flgA*	Flagellar basal-body P-ring formation protein FlgA	Basal-body P-ring biosynthesis	BMEII1085	BRA1055	BAB2_0152	229	3274 (97%)	917 (99%)	1453 (98%)
	*flgI*	Flagellar P-ring protein FlgI	P-ring monomer	BMEII1084	BRA0156	BAB2_0154/0153	230	3273 (99%)	916 (99%)	1452 (99%)
III	*fliG*	Flagellar motor switch protein FliG	Motor switch	BMEII1113	BRA0122	BAB1_0121	202	3302 (99%)	945 (99%)	150 (99%)
	*fliM*	Flagellar motor switch protein FliM	Motor switch	BMEII1110	BRA0125	BAB1_0125/0124	205	3299 (97%)	942 (98%)	147 (97%)
	*flgF*	Flagellar basal-body rod protein FlgF	Basal-body rod	BMEII1107	BRA0128	BAB2_0128	208	3296 (98%)	939 (98%)	144 (99%)
	*fliI*	Flagellum-specific ATP synthase FliI	ATP synthase	BMEII1105/1106	BRA0129	BAB2_0129	209	3295 (99%)	938 (99%)	143 (99%)

Flagella related genes predicted to be pseudogenes in B. melitensis, B. suis, and B. abortus were compared to their counterparts in B13-0095, BO1, and BO2 genomes. The values in brackets are the % of identity at the protein level when compared with the corresponding gene in B13-0095 genome. Pseudogenes are shown in gray boxes.

### The Pac-Man frog *Brucella* isolate produces an atypical LPS

**Figure 4:**
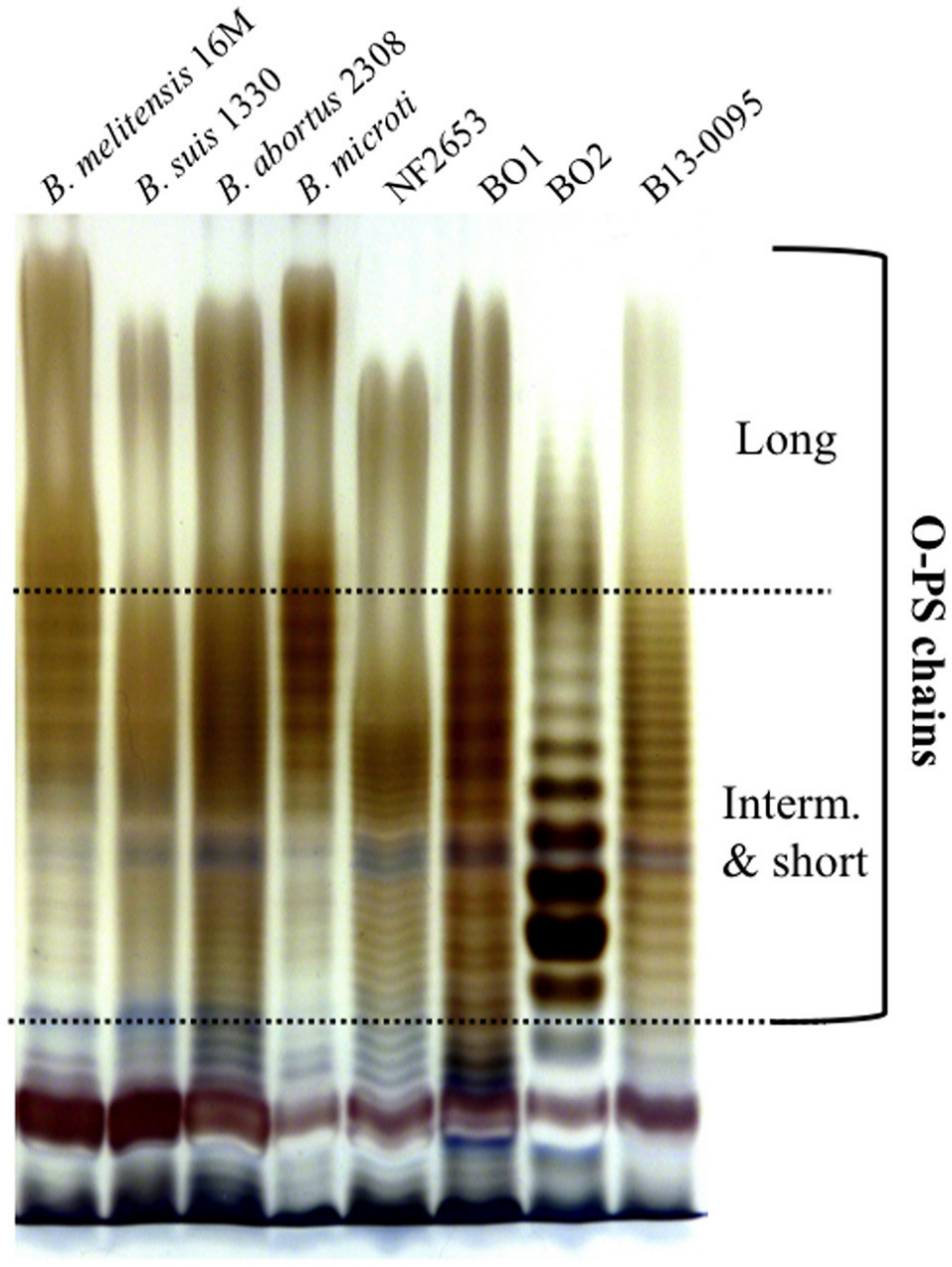
**Electrophoretic profiles of the LPS produced by differen*t Brucella***. Silver staining after SDS-PAGE of proteinase K-digested LPS preparations from the indicated *Brucella* strains. The bands corresponding to the long, intermediate or short sugar chains of O-PS are indicated.

As reported for the African bullfrog isolates (Eisenberg et al., 2012), B13-0095 does not agglutinate with *Brucella* specific sera. This suggested that, as seen for BO2, this strain does not synthesize the typical *Brucella* perosamine based O-antigen (Wattam et al., 2012). We thus analyzed the LPS produced by B13-0095 by SDS-PAGE and silver staining and compared the pattern obtained to that of BO1, BO2, or NF2653, as well as smooth classical *Brucella* strains (Figure 4). The O-antigen of B13-0095 displayed a continuous ladder-like pattern typical of a smooth LPS in which O-antigen is made of repeated oligosaccharide units. This pattern, with very close and regularly spaced narrow bands, looked similar to that observed with BO1 and NF2653. However, contrary to the other smooth LPS displaying a classical bimodal distribution (i.e., containing both long- and intermediate-chains of polysaccharides), the B13-0095 O-antigen seems to be predominantly composed of short- and intermediate-chains of polysaccharide.

**Figure 5:**
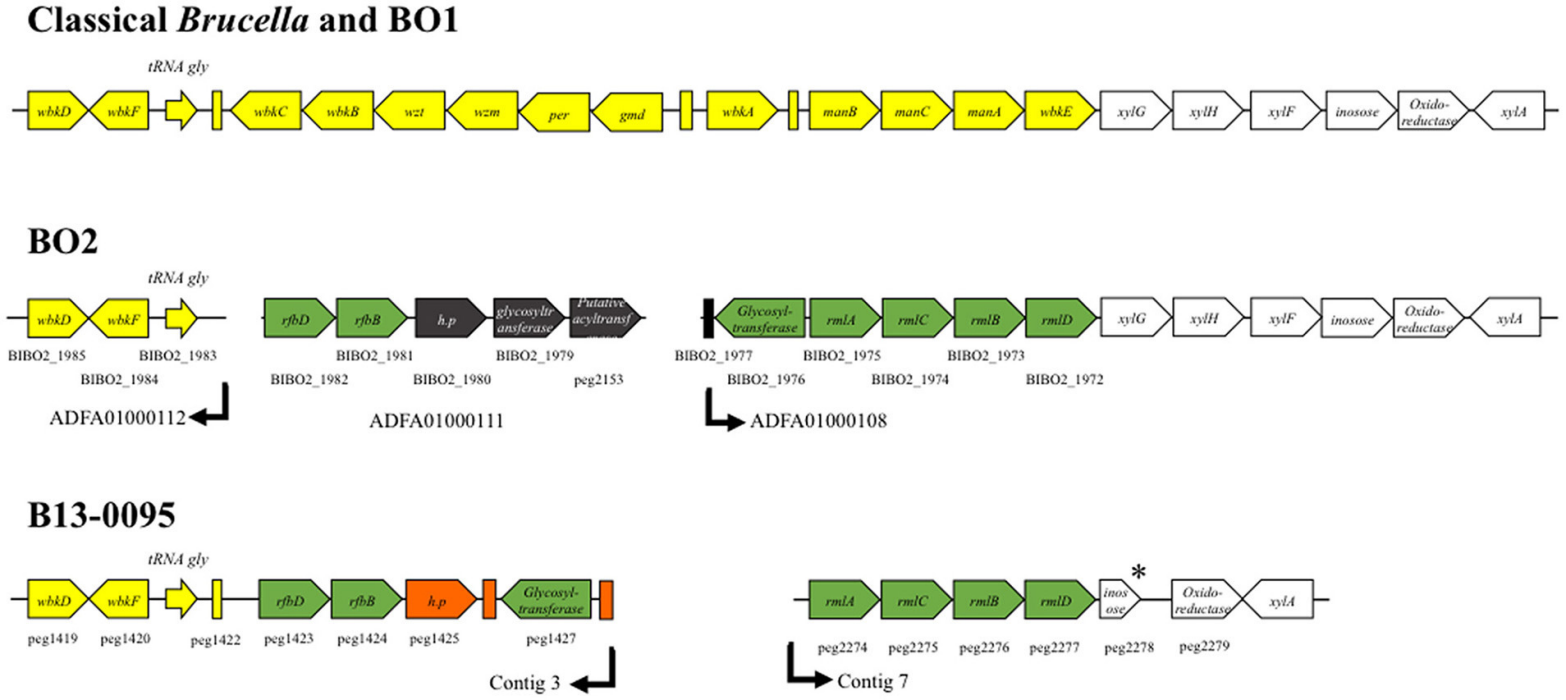
**Schematic representation of the wbk region organization in *Brucella***. The genes described in classical *Brucella* and *B. inopinata* BO1 within or flanking the wbk region are shown in yellow or white, respectively. The genetic features present in both the Pac-Man frog isolate B13-0095 and the *B. inopinata*-like strain BO2 genomes are shown in green. Features in black are only present in BO2 and features in orange only in B13-0095. The annotation of the genes is indicated, as well as their ID number in PATRIC (peg#) or RefSeq (BIBO2#). The arrows indicate the end/beginning of the contigs in the draft genome sequences of B13-0095 and BO2. ^*^ indicate predicted pseudogenes. Rectangles represent predicted mobile elements.

We then analyzed the genes involved in LPS biosynthesis in the B13-0095 genome, focusing first on the *wbk* and *wbo* regions that contain the genes involved in O-polysaccharide synthesis in classical *Brucella*. We found that, like BO2, B13-0095 lacks the *wboA* and *wboB* genes (Table S4). The organization of the *wbk* region is also similar to that in BO2, with 11 missing genes that are replaced by several others. Four of them (*rmlA, rmlC, rmlB*, and *rmlD*) are used in some bacteria for making a rhamnose-based O-antigen (Figure 5 and Table S4). The others encode a glycosyltransferase, a hypothetical protein and two genes (*rfbB* and *rfbD*) predicted to be involved in O-antigen export but that differ from those with the same function in the classical *Brucella* (*wzm* and *wzt*). It should be noted that the genes BIBO2_1979 to BIBO2_1982 in the *wbk* region of BO2 were not described by Wattam et al. (2012), probably because they are contained in a small contig of 2.14 kb (contig ADFA01000111). Another feature specific to B13-0095 is the lack of three genes (*xylG, xylH*, and *xylF*) that are found in the immediate vicinity of the *wbk* region in classical *Brucella* and predicted to encode a xylose import system. The genes encoding the enzymes involved in the modification of sugar precursors (*pgm, manB*_core_, *manC*_core_, *kdsA*, and *kdsB*) or incorporation of sugars (*wadA, wadB*, and *wadC*) into *Brucella* LPS are present in B13-0095 genome. All the genes involved in lipid A biosynthesis are also present in B13-0095 genome, however region 2 contains an additional fragment of 6.5 kb encoding hypothetical or phage proteins. Finally, PATRIC identified several other genes putatively related to LPS in B13-0095 genome, all of them being present in all the *Brucella* analyzed in this study. Among them, *lptA, lptB*, and *lptC* are predicted to encode proteins involved in LPS export to the outer membrane. The Lpt system is encoded by 7 genes in *E. coli* (Sperandeo et al., 2009); search for orthologs identified the genes encoding the 4 remaining proteins, which are conserved across *Brucella*.

### The Pac-Man frog *Brucella* isolate has a fast replication rate within eukaryotic cells

**Figure 6:**
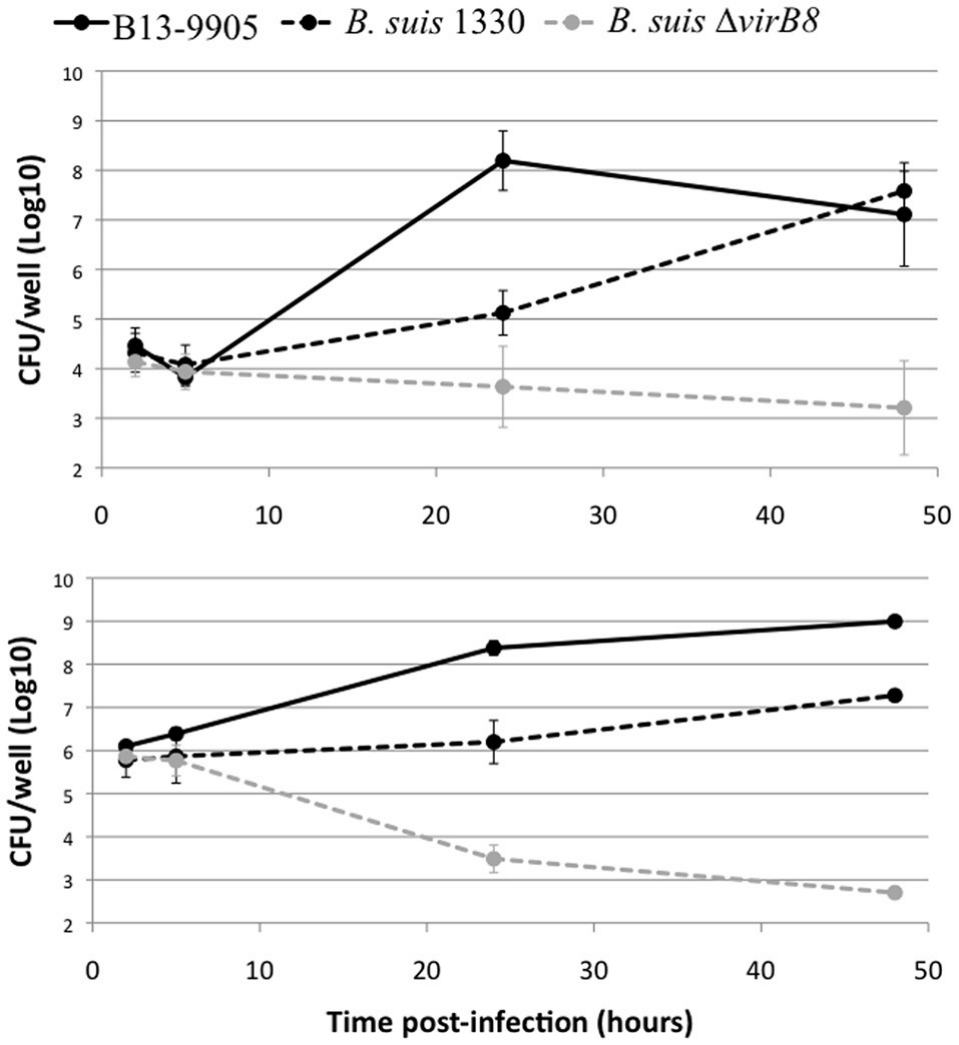
**Intracellular replication profile in eukaryotic cells**. Intracellular replication was determined using gentamycin protection assays. HeLa (top) or J774 (bottom) cells were infected with *B. suis* bv.1 wild-type (black dotted line), a *B. suis virB8* mutant (gray dotted line), or the frog isolate (full line) and lysed at 2, 5, 24, or 48 hpi. The data are shown as Log_10_ CFU/well and represent the geometric mean for three independent experiments done in triplicate.

*Brucella* are facultative intracellular pathogens. Their ability to replicate inside eukaryotic cells, the major feature for their pathogenic potential, is highly dependent on the structure of their LPS (Porte et al., 2003; Mancilla, 2016). We thus measured the intracellular replication rate of B13-0095 in human epithelial cells (HeLa) and murine macrophages (J774), compared to *B. suis* strain 1330, with replication and intracellular trafficking profiles that are well characterized. We found that B13-0095 grows much faster within both HeLa and J774 cells (Figure 6). At 24 h post-infection (hpi), the intracellular bacterial numbers are 100–1000 times higher than observed with *B. suis* in HeLa or J774 cells, respectively, and reminiscent of that observed with BO1 (Jiménez de Bagüés et al., 2014). The *B. suis* Δ*virB8* mutant provides a control showing that both cell lines are able to control infection by attenuated strains. Thus, the elevated rate of replication of the frog isolate cannot be attributed to a reduced fitness of these cells.

**Figure 7:**
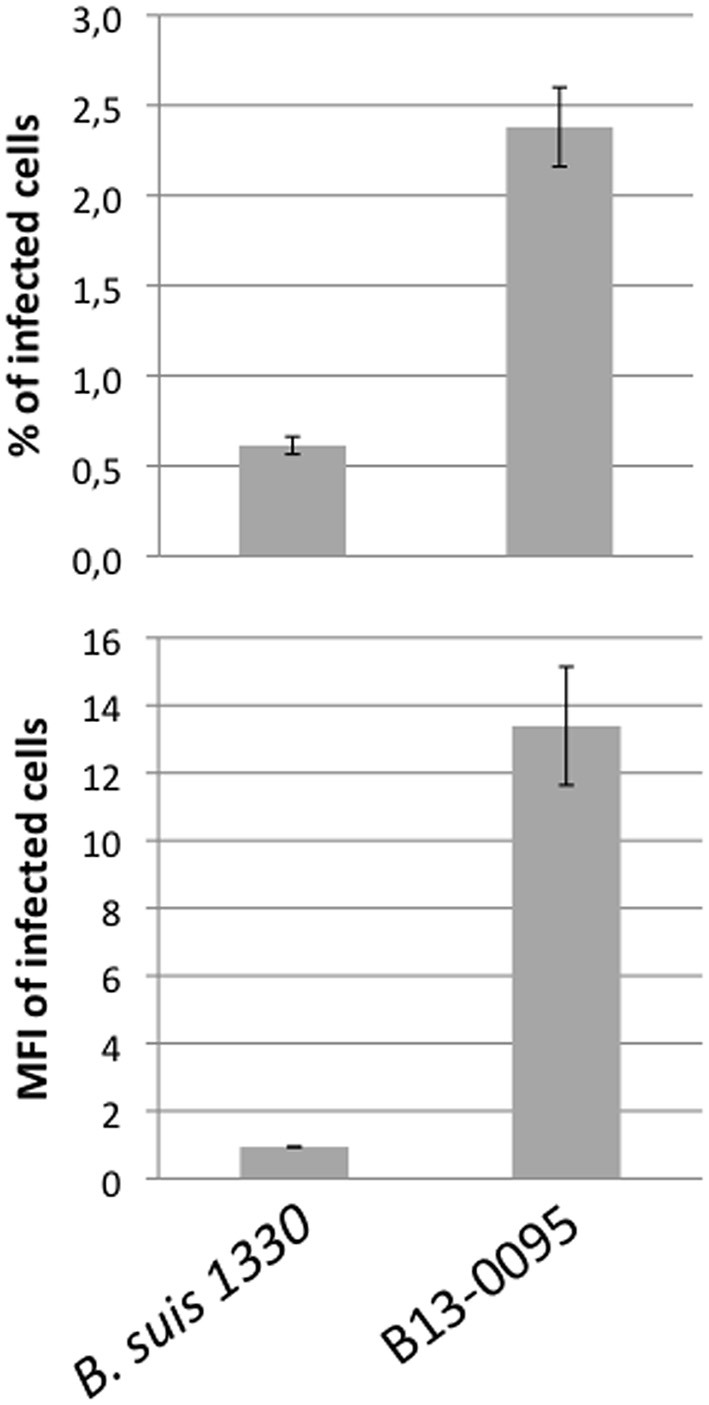
**Quantification of infection rate by flow cytometry**. HeLa cells were infected by mCherry-expressing bacteria, detached, and fixed 24 hpi and analyzed by flow cytometry. The level of infection is given by the % of infected (i.e., mCherry^+^) cells (top). The fluorescence intensity of infected cells was quantified by the MFI (Median Fluorescence Intensity) values (bottom), which is indicative of the intracellular replication rate of the bacteria. The data are mean values (±SEM) calculated on 3 replicates.

**Figure 8:**
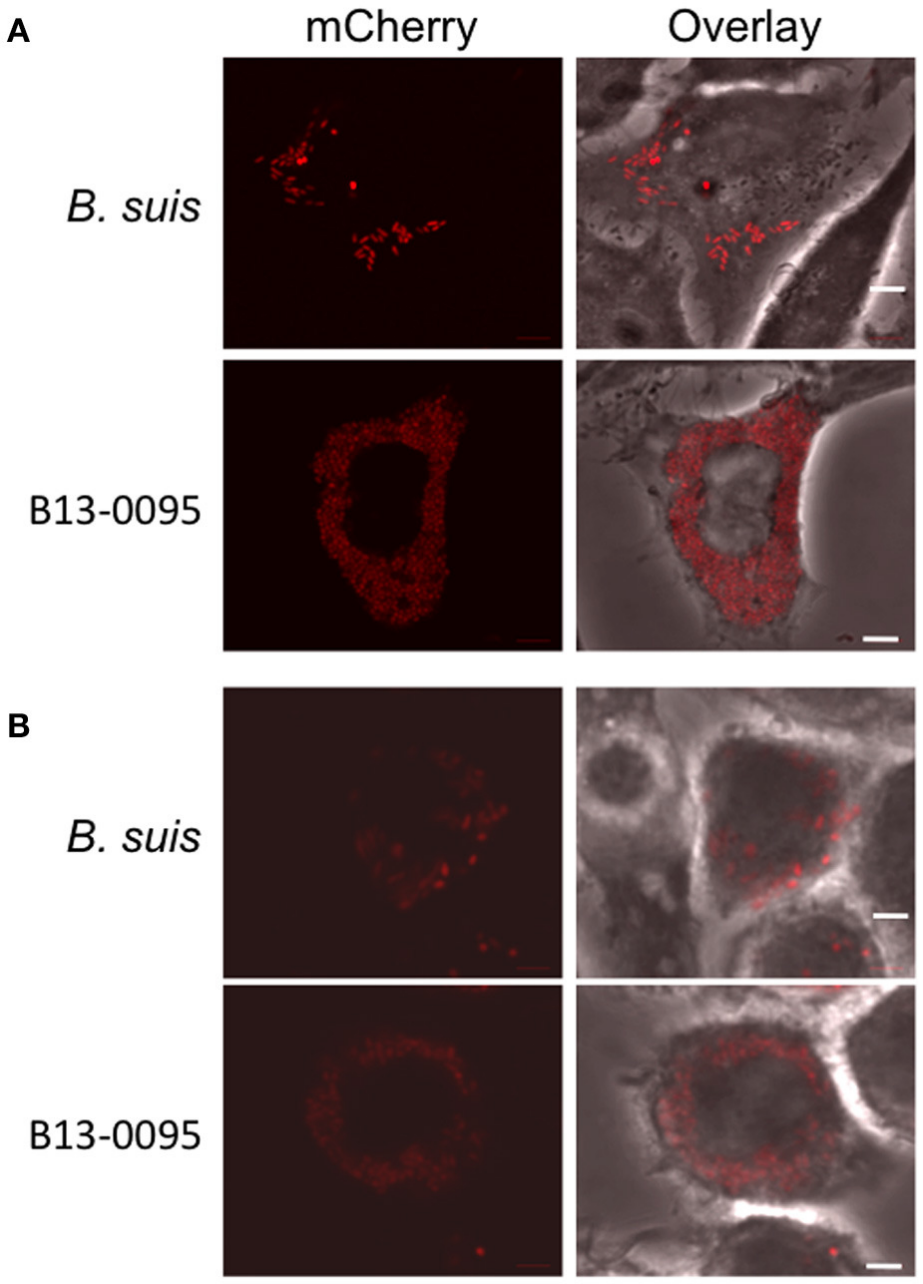
**Visualization of B13-0095 intracellular replication**. HeLa **(A)** or J774 **(B)** cells were infected by bacteria expressing mCherry, fixed at 24 hpi and visualized by confocal microscopy using a 60x objective. Shown are representative images for each condition. Bacteria are shown in red. Cells are visualized by transmission in the overlay. Scale bar = 5 μm.

Low magnification observation of cells infected with fluorescent bacteria at 24 hpi suggested that, with B13-0095, there were more cells infected and that infected cells contained more bacteria (data not shown). This was confirmed using a flow cytometry analysis in which the level of infection is given by the % of fluorescent cells and the rate of intracellular replication is given by the median fluorescence intensity (MFI) of infected cells. We observed that B13-0095 infects more HeLa cells than *B. suis* and replicates much faster within infected cells (Figure 7). High-resolution observations of infected cells revealed many more bacteria at 24 hpi with the frog isolate than with *B. suis* (Figure 8). At 24 h pi, the cytoplasm of cells infected with B13-0095 were already fully packed with bacteria, while this is usually observed after 48 hpi with *B. suis*. Identical observations were made with BO1, but not with BO2 (K. Garcia-Mendez and A. Keriel, unpublished data). B13-0095 is more prolific with regard to infection and replication within eukaryotic cells than classical *Brucella*.

## Discussion

### Potential sources of infection in frogs

Over the first years of the 21st century the *Brucella* genus has expanded from 6 to 11 known species, all of which were associated with mammals. In 2012, the natural hosts range expanded to amphibians after the reports of atypical strains from frogs. The first cases described in Germany (Big-eyed tree frog and African bullfrogs, Eisenberg et al., 2012; Fischer et al., 2012) were in animals imported from Africa, while the White's tree frog in the UK belongs to a species originally native to Australia (Whatmore et al., 2015). B13-0095 is the first *Brucella* strain isolated from an amphibian indigenous to the Americas.

In the Big-eyed and White's tree frogs, bacteria were isolated from superficial lesions that did not affect the animal's overall health, whereas the African bullfrogs were found dead or moribund, similar to the Pac-Man frog described in this study. All these frogs were captive, being either pets (Big-eyed tree and Pac-Man frogs) or part of tropical animal collections (White's tree frog and African bullfrogs), and had limited contacts with exterior environment. The actual sources of the infections, or whether the animals were infected in the wild or during their time in captivity, are unknown. Possibly the mice used to feed the big frog species (Pac-Man and African bullfrogs) could have been the source of a foodborne, fatal infection. In this respect, the White's and Big-eyed tree frogs, that had only superficial infections, are not fed with mice but with insects or worms. Alternatively, the bacteria could be a contaminant in the substrates added in the terrarium of the animals, such as coco husk, bark chippings, or plants, or in live moss, which is often used to maintain housing humidity. Some of the specific genetic features of the Pac-Man frog isolate B13-0095 are shared with bacteria of the rhizosphere (see below), suggesting that these bacteria could be associated with plants roots or found in the soil.

### Early-diverging *Brucella* as an intermediate stage between a soil associated ancestor and host adapted species

Analysis of *Brucella* genome sequences has begun to unearth the pathway of evolution from an environmental organism to a stealth pathogen. This involved acquisition of genetic material encoding virulence factors combined with genome reduction (Wattam et al., 2014b). Phylogenetically, *Brucella* divide into two clades, the “classical” *Brucella* and an early dividing branch of atypical strains. The kSNP analysis (Figure 1) shows that B13-0095 belongs to the group of early-diverging brucellae with traits that depart significantly from those of the classical *Brucella* spp. This group includes the two strains that form the “BO clade” (*B. inopinata* BO1 and strain BO2), a group of strains from Australian rodent strains and the previously reported frog isolates. MLST analysis has highlighted the genetic heterogeneity among the frog isolates (Eisenberg et al., 2012; Whatmore et al., 2015), suggesting that B13-0095 may also form a separate branch in this group. Phylogenetic placement of B13-0095 compared to the other frog isolates using WGS data will help understanding the evolutionary and phylogenetic relationships among the amphibian isolates.

Several features in B13-0095 genome suggest that it represents another stage in the evolution of *Brucella* from a soil associated ancestor to the host adapted “classical” species. Two regions endow metabolic capacities associated with soil bacteria that the “classical” *Brucella* have lost. An ectoine utilization gene cluster, conserved in *O. antropi* and some environmental halophilic bacteria, allows B13-0095 to use ectoine as a carbon source. Another genetic region (already noted in Wattam et al., 2012), which is conserved within the BO clade *Brucella* and *O. anthropi*, contains genes encoding rhamnose uptake and utilization systems. The genes are organized as those on a plasmid from *R*. *leguminosarum*, where this locus has been extensively studied (Oresnik et al., 1998). We found that B13-0095, BO1, and BO2 (but no other *Brucella)* have the ability to use rhamnose as a carbon source. This may be linked to the closer proximity of the BO clade *Brucella* to environmental ancestors. Rhamnose is commonly found in plants as a part of complex pectin polysaccharides. These compounds can be degraded by saprophytic or plant-pathogenic bacteria using a set of extracellular enzymes (Rodionova et al., 2013). The resulting plant-derived rhamnose can then be used as a carbon source by microorganisms in the rhizosphere. We did not find any genes involved in the degradation of plants compounds in B13-0095 genome, suggesting that, if these bacteria can reside in the rhizosphere, they would only be able to use rhamnose made available by other organisms in this symbiotic community.

### Atypical O-PS in *Brucella*: role in virulence?

**Figure 9:**
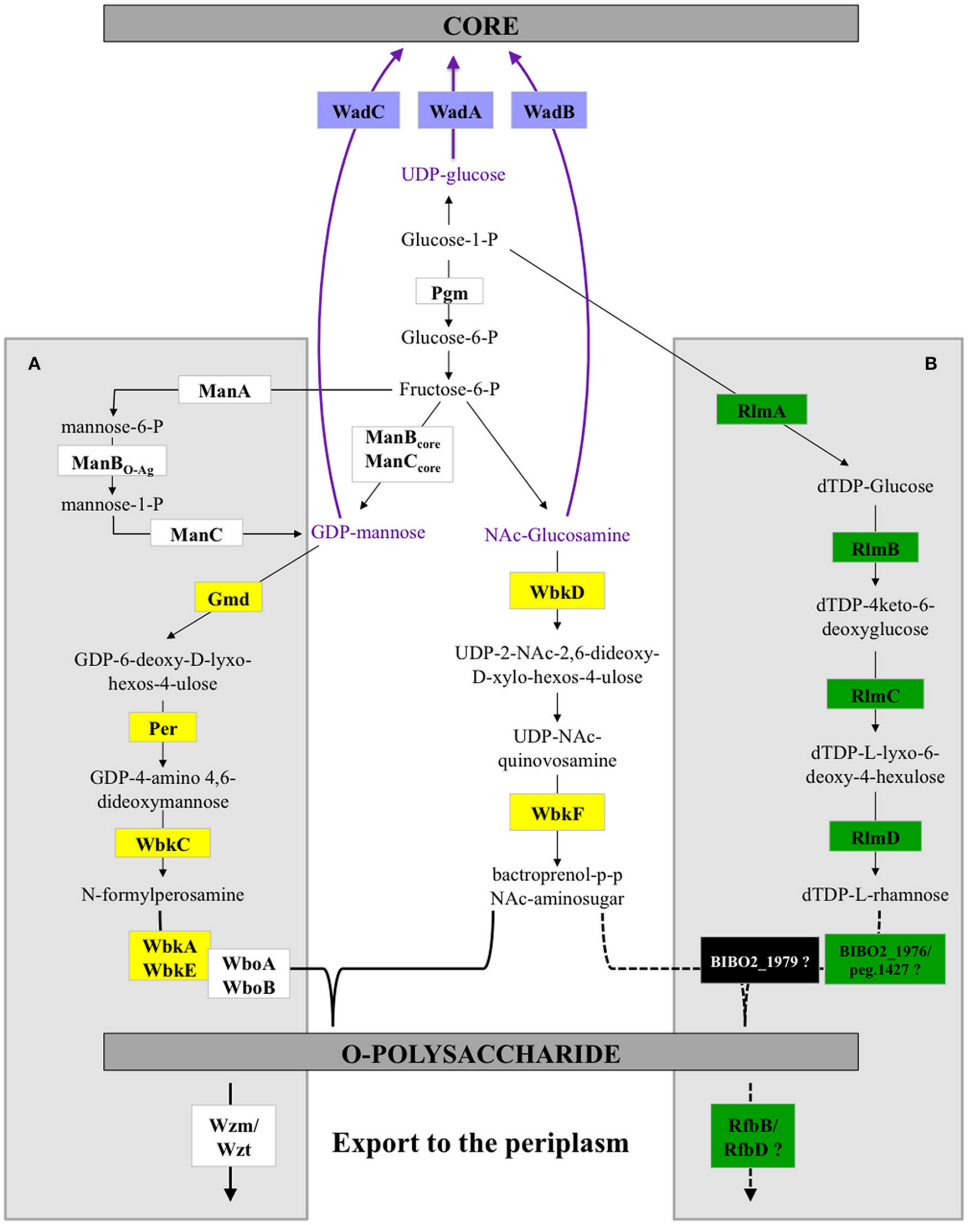
**LPS biosynthesis pathways in *Brucella***. Schematic representation of the O-polysaccharide biosynthesis pathways in *Brucella*. The A box highlights the reactions previously described for classical *Brucella.* The B box shows the predicted alternative pathways in the Pac-Man frog isolate B13-0095 and strain BO2. The reactions outside the boxes are common. Dotted lines show speculated reactions. The color code used to highlight some enzymes in this scheme is the same as in Figure 5 and Table S4.

Our bioinformatics analysis has highlighted novel features concerning the genetics of LPS synthesis in B13-0095 and has also given new information on the LPS export applicable to all *Brucella.* With the exception of the two naturally rough species (*B. canis* and *B. ovis*) *Brucella* were traditionally reported to have a perosamine based O-antigen. In the classical *Brucella*, mannose is used as a sugar precursor for the synthesis of N-formylperosamine, which is then transferred to a bactoprenol molecule by several glycosyltransferases (WbkA, WbkE, WboA, and WboB) (Figure 9). The O-PS is then exported from the cytoplasm to the outer leaflet of the inner membrane (periplasm) using a system encoded by *wzm* and *wzt*. We found that, as with BO2, B13-0095 lacks many of the genes of the *wbk* region involved in the synthesis of this perosamine-based O-PS and has instead genes that several plant-associated bacteria use for making a rhamnose-based O-PS (Jofré et al., 2004; Broughton et al., 2006; Balsanelli et al., 2010; Clifford et al., 2013). B13-0095 and BO2 possess all the enzymes required to synthesize dTDP-rhamnose from glucose. This modified sugar may then be transferred to bactoprenol by the putative glycosyltransferases encoded by the genes BIBO2-1976/peg.1427, BIBO2-1980, or by other glycosyltransferases encoded elsewhere in their genomes. The O-PS could then be translocated to the periplasm using RfbB and RfbD, both predicted to be involved in O-antigen export.

LPS is considered as a major virulence factor in *Brucella* (Porte et al., 2003). The structure of the core plays a role in the ability to avoid recognition by the innate immune response (Fontana et al., 2016) and a complete, perosamine based O-PS has been shown to be essential for host-pathogens interactions for the smooth classical *Brucella*, being involved in an interaction with lipid rafts at the cell surface that allow bacteria to enter and traffic through a pathway targeting them to an appropriate intracellular replicative niche (Porte et al., 2003). The low intracellular replication rate of *Brucella* strain BO2 (B. Saadeh and D. O'Callaghan, unpublished data), which has a rhamnose based O-antigen, led to the suggestion that the acquisition of a perosamine based LPS was one of the key steps in the evolution of *Brucella* virulence (Wattam et al., 2012). However, the fast intracellular multiplication of B13-0095 shows that other factors are also important. Future work will be required to characterize biochemically the O-antigen produced by B13-0095 and determine its role in virulence.

After linking the O-PS to core-lipid A in the periplasm, the assembled LPS molecules must be shuttled to the outer membrane. In several Gram-negative bacteria, the lipopolysaccharide transport (Lpt) machinery is responsible for the export of LPS from the periplasmic surface of the inner membrane, across the periplasm, to the outer leaflet of the outer membrane (Sperandeo et al., 2009). The seven proteins in the Lpt system (LptA to LptG) constitute a large complex spanning the inner to the outer membrane and operating as a concerted machine. The identification of all the genes encoding the Lpt export system in *Brucella* adds another step in our comprehension of LPS synthesis in this genus.

### The BO-clade *Brucella* are motile

Classically, *Brucella* are classified as non-motile bacteria. One of the major finding in our study is the demonstration that the atypical BO clade *Brucella*, including B13-0095, BO1, and BO2, are highly motile. Motility among *Brucella* isolates from African bullfrogs was reported (Eisenberg et al., 2012) however, as no data was presented, it is not possible to compare motility efficiencies among these strains. Importantly, it should be noted that the tests performed in agar tubes upon initial isolation of B13-0095 in Texas did not allow detection of the motility of this organism, suggesting that these tests, commonly used for biotyping, may not be optimal for some bacterial species. In this respect, the initial reports on BO1 and BO2, which probably also used in tube assays, describe both strains as non-motile (De et al., 2008; Tiller et al., 2010a).

During evolution, the classical *Brucella* have accumulated mutations that have led to the formation of several pseudogenes among flagella related genes (Letesson et al., 2002; Chain et al., 2005; Tsolis et al., 2009). Despite these mutations, under specific growth conditions, these strains can make a sheathed flagellum that does not confer motility, but is required for virulence (Fretin et al., 2005; Petersen et al., 2011). Analysis of the flagella genes in B13-0095, BO1, and BO2 showed that all the genes were fully functional, probably explaining the motility of these three strains. The second group of atypical strains, isolated from Australian rodents and represented by NF2653, are non-motile and also have flagella pseudogenes. This gives us a starting point for the process of flagella degradation. Motility in the BO clade *Brucella* is probably a residual property from environmental ancestors that could provide a strong advantage by allowing it to move to a favorable niche. Understanding the role of this motility in the virulence of these strains and their intracellular lifestyle will require further studies. Motility could provide an advantage at different stages of their pathogenic cycle: reaching the host, invading and colonizing the host cells, and dispersing to new hosts. At the cellular level, motility could help the bacteria reach a permissive cell or entry site, as it has been shown for *Salmonella* that “near surface-swimming” motility enhances bacterial uptake (Misselwitz et al., 2012). A role for motility in reaching the host cell surface would be consistent with an elevated rate of infection observed for B13-0095 (this study), BO1 and BO2 (B. Sadeeh and D. O'Callaghan, unpublished data). It would also be of interest to determine whether the FliC flagellin encoded by the BO clade *Brucella* lacks the TLR5 antagonist property demonstrated in *B. melitensis* (Terwagne et al., 2013).

### Risks of misidentification of atypical *Brucella* isolates

Accurate identification of pathogens is essential for establishing dependable diagnosis, choosing a treatment, and understanding the source of infection. This study highlights the risk that atypical *Brucella* spp. isolated from amphibians may be misidentified as *O. anthropi*, an opportunistic pathogen in humans, particularly in immunocompromised patients, which has been associated with wound infections, abscesses or septicemia (Kettaneh et al., 2003; Ozdemir et al., 2006; Vaidya et al., 2006; Hagiya et al., 2013). *O. antropi* is widely distributed in the environment (water, plants, soil) and can contaminate indwelling medical devices. Infections with *O. anthropi* have also been reported in free-ranging amphibians (cane toads) in Australia (Brown et al., 2007; Shilton et al., 2008). Despite being *bona fide Brucella*, the frog isolates were not identified as such. First, the rapid growth rate excluded the identification as *Brucella* as did the lack of agglutination with specific antisera. In many clinical laboratories, the initial identification is made using the commercially available tests such as API 20NE. However, we and others found that API 20NE tests misidentify fast growing atypical *Brucella* spp. such as *O. anthropi* with a very high confidence level (Scholz et al., 2010; Tiller et al., 2010a; Fischer et al., 2012). Thus, in all human and animal cases in which identification of the pathogen as *O. anthropi* was solely based on this test (including the cane toads cited above), involvement of atypical *Brucella* spp. cannot be excluded. Matrix assisted laser desorption/ionization-time of flight (MALDI-TOF) mass spectrometry (MS) is rapidly becoming the method of choice for bacterial identification in clinical laboratories. It allows a fast identification of bacteria and yeast, but its accuracy largely depends on the coverage of the database. With regard to *Brucella*, accurate identification by MALDI-TOF MS is currently limited because this genus is either not represented in the databases of the two main manufacturers, or only in a bioterrorism database with restricted availability (Cunningham and Patela, 2013). Using a safe inactivation protocol developed recently (Mesureur et al., 2016), we constructed an exhaustive MALDI-TOF MS *Brucella* database that can identify all *Brucella* spp., in most cases at the species level (J. Mesureur and A. Keriel, unpublished data). This database allowed us to clearly discriminate the frog isolates B13-0095 from *O. anthropi* and identified it as *B. inopinata* (this data will be presented in a separate publication). Another possibility would be to exploit the absence of some genes (shown in red in Figure 2 and Table S3) from *Ochrobactrum* spp. genomes to discriminate between atypical *Brucella* and *O. anthropi* using PCR.

Isolation of *Brucella* spp. from frogs from Africa, Australia and America suggests that they may be widespread and highlight a need for a broader assessment of the presence of *Brucella* in amphibians worldwide. Thus, not only better identification tools are required, but prevention measures should also be taken. Whilst the zoonotic potential of this group is not known yet, their close proximity with strains associated with human disease suggests that they might present a risk to the animals' keepers and thus, unnecessary contact with potentially infected amphibians should be avoided.

## Authors contributions

PS, CQ, DO, and AK designed the study; PS, SL, TS, JE, and AK acquired the data; PS, CQ, SL, TS, DO, and AK analyzed and interpreted the data; PS, CQ, SL, JE, TF, SR, DO, and AK participated in drafting the article or revising it critically.

## Funding

This work was supported by the Institut National de la Santé et de la Recherche Médicale (INSERM), Université de Montpellier and the Institut de Veille Sanitaire (InVS). PFSL is recipient of a post-doctoral research grant from the Fondation Méditerranée Infection (France).

### Conflict of interest statement

The authors declare that the research was conducted in the absence of any commercial or financial relationships that could be construed as a potential conflict of interest.

## Footnotes

^1^https://www.patricbrc.org

^2^http://blast.ncbi.nlm.nih.gov

^3^http://imagej.net/

^4^https://www.patricbrc.org
