# Supplementary material for: A Brucella spp. Isolate from a Pac-Man Frog (Ceratophrys ornata) Reveals Characteristics Departing from Classical Brucellae
Source: Front Cell Infect Microbiol. 2016 Sep 28;6:116. doi: 10.3389/fcimb.2016.00116 (PMC5040101; doi:10.3389/fcimb.2016.00116)
Supplement: Supplementary file 2 [file DataSheet2.DOCX]

Supplementary Material

**Characterization of an Atypical *Brucella* Isolate from a Pac-Man Frog (*Ceratophrys ornata*) Reveals New Metabolic Pathways and a Remarkable Motility within this Bacterial Genus**

Pedro F. Soler-Lloréns ^1,2^, Chris R. Quance ^3^, Sara Lawhon ^4^, Tod P. Stuber ^3^,

John Edwards ^4^, Thomas A. Ficht ^5^, Suelee Robbe-Austerman ^3^, David O’Callaghan ^1,2^ and Anne Keriel ^1,2,*^

^*^ **Correspondence:**

Anne Keriel

[anne.keriel@inserm.fr](mailto:anne.keriel@inserm.fr)

**LEGENDS TO SUPPLEMENTAL TABLES**

**Table S1 :** List of genomes used for the kSNP analysis in Figure 1.

**Table S2:** List of the predicted protein encoding genes conserved in the BO clade *Brucella* (ie BO1, BO2 and B13-0095) and, in some cases in O*. anthropi* ATCC49188. +/- indicate whether the gene is present or not in the corresponding genome. The genes highlighted in blue belong to the rhamnose utilization cluster described in Figure 2B.

**Table S3:** Organization of the L-Rhamnose genes cluster (in blue) and of the 3 loci of the flagellar related genes in *B. suis* 1330, BO1, BO2 and B13-0095, as well as in *O. anthropi* ATCC49188.

**Table S4:** Analysis of LPS related genes in B13-0095. All the genes annotated in PATRIC as related to LPS in the B13-0095 genome were compared to the genes in BO1 and BO2, as well as in *B. melitensis* 16M, *B. suis* 1330 and *O. anthropi* ATCC49188. Absent genes are highlighted as grey boxes. * indicate pseudogenes. The same color code was used in this table and in Figures 5 and 9.
